# An Overview on Lipid Nanocapsules: Exploring the Role in Precision Cancer Treatment and Lymphatic Drug Distribution

**DOI:** 10.34172/apb.025.45109

**Published:** 2025-06-01

**Authors:** Mahesha Keerikkadu, Pragathi Devanand Bangera, Vamshi Krishna Tippavajhala, Mahalaxmi Rathnanand

**Affiliations:** Department of Pharmaceutics, Manipal College of Pharmaceutical Sciences, Manipal Academy of Higher Education, Manipal 576104, India

**Keywords:** Lipid nanocapsule, Tumor targeting, Cancer, Systemic toxicity, Ligands

## Abstract

Lipid nanocapsules (LNCs) are an emerging nanocarrier platform for cancer therapy as they can co-deliver multiple drugs, promote synergistic action, and provide targeted drug delivery. The phase inversion temperature (PIT) process is most used for LNC formulation, which has the advantage of process simplicity, thermodynamic stability, and the employment of non-toxic solvents without requiring high energy input. Surface functionalization with targeting ligands like folic acid and peptides increases tumor specificity and reduces off-target toxicity. The nanoscale dimensions and stealth properties of LNCs also take advantage of the enhanced permeability and retention (EPR) effect for enhanced tumor accumulation. LNCs provide precise cancer therapy through the ability to deliver drugs selectively, improve bioavailability, and reduce systemic toxicity. Their nanometer dimensions and surface characteristics allow for effective lymphatic uptake and passive tumor targeting. LNCs offer a potential platform for site-specific treatment, particularly in metastatic cancer with lymphatic involvement. LNCs have become multifunctional platforms with accurate, effective, and patient-friendly delivery systems for cancer treatments. This review critically examines new developments in LNC-based cancer therapies, focusing on optimization of physicochemical properties, improved targeting efficiency, and facilitation of combination therapy. In addition, it draws attention to the translational advantages of LNCs in alleviating systemic toxicity, enhancing pharmacokinetics, and overcoming multidrug resistance in cancer treatment.

## Introduction

 Cancer is multifaceted and multidisciplinary, representing a huge disorder of unregulated growth with the propagation of abnormal cells within the body. Malignant tumors may infiltrate the surrounding tissues through metastasis, spreading the disease elsewhere in the body using blood or the lymphatic system.^1^ The lymphatic system’s role in these tumors underscores the complex relationship between the immune system and the development of cancer, underscoring the urgent need for tailored therapeutic strategies for every kind of lymphoma.^2^ Filtering and moving lymph, a fluid containing white blood cells that fight infections, is a vital function of the lymphatic system and a component of the body’s immune system. But much like other bodily systems, it can turn into a place where cancer starts and grows. Primary lymphatic system malignancies are lymphomas and blood cancers impacting the body’s immune system. The lymphatic system, which includes the spleen, thymus, bone marrow, and lymph nodes, is essential to the body’s defense systems.^3^ Leukemia is a blood cancer that originates in the lymphatic system. It is primarily derived from the bone marrow and only indirectly affects the blood and other organs.^4^ Hodgkin lymphoma and non-Hodgkin lymphoma are the two primary forms of lymphomas. The disease’s characteristic, giant aberrant cells known as Reed-Sternberg cells, set Hodgkin lymphoma apart. This kind of lymphoma typically starts in the lymph nodes and can spread to other organs or areas of the lymphatic system. Conversely, non-Hodgkin lymphoma comprises all forms except Hodgkin lymphoma, making it a more varied category of blood malignancies.^5^

 Chemotherapy involves inhibiting tumor cells or stopping the growth and multiplication of tumor cells using chemical agents.^6^ However, existing cancer therapy has several side effects affecting the quality of life.^7^ The disadvantages of currently available cancer medications are the non-specific targeting, short biological half-lives, and adverse responses that can affect therapy response and clinical outcome; they may also inhibit regular cell activity and induce resistance.^8^ The nanoparticle-based drug delivery systems in cancer treatment have provided various benefits, such as improved pharmacokinetics, targeted inhibition of tumor cells, reduced side effects, and reduced drug resistance.^9^ Polymer and lipid nanoparticles (LNPs) have mainly been utilized in designing and developing anticancer drug delivery.^10^ The LNP system is an advanced method for treating cancer, including different groups of versatile lipids forming in a stable core-shell pattern. This creates numerous voids in the medicinal substance during the inclusion process by encapsulation. It attracts those substances toward various destinations across the hydrophilic or hydrophobic parts, thus enhancing their solubility, stability, and bioavailability. Their biocompatibility and biodegradability reduce the risk associated with toxicity from traditional drug delivery systems. These nanoparticles are helpful for specific applications in therapy.^11,12^ Different LNPs, including hybrid lipid-polymer nanoparticles,^13^ liposomes,^14^ nanostructured lipid carriers,^15^ exosomes,^16^ cubosomes,^17^ solid LNPs,^18^ and nanocapsules (NCs)^19^ have been used for cancer research, targeting, and treatment. These formulation’s weaknesses lessen the efficiencies of cancer treatment: low dissolving rate, shortened circulation time, no stability in the stomach, low drug loading capacity, and lipid destruction in storage.^20^

**Figure 1:**
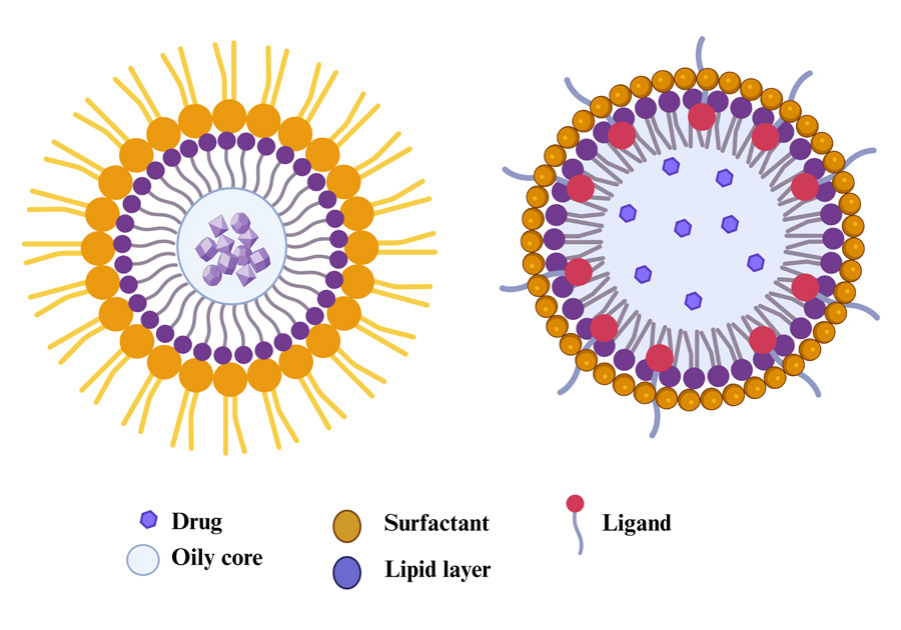
Structural features of lipid nanocapsules

 The special properties of lipid nanocapsules (LNCs) make them different from all other nanocarriers. As illustrated in Figure 1, LNCs are composite structures that trap drugs into a lipid or polymer membrane cavity. The outermost enveloping layer that encloses the oily core is termed LNCs or polymeric nanocapsules.^21^ LNCs result from the hybridization between liposomes and polymeric nanoparticles. LNCs are more potent for the encapsulation of drugs and biocompatible in comparison to traditional nanoparticles.^22^ Even though liposomes are the most researched nanocarrier used for the encapsulation of drugs, they have several severe drawbacks, one of which is that their encapsulation capacity for lipophilic drugs is very low. Organic solvents used in its production are also volatile and liable to leak into aquatic environments and biological fluids.^23^ LNCs have a smaller particle size than the endothelium fenestrations. LNCs are also superior to LNP. The research on LNCs is in progress to overcome the constraints of LNPs.^24^ Lipid nanocarriers are favorable to be used instead of liposomes to encapsulate lipophilic molecules because of their outstanding encapsulation efficiency. The hybrid structure of the LNCs favors the functionalities of both polymer NCs and liposomes. LNCs are produced via soft-energy techniques that exhibit outstanding stability without leakage of formulations.^25,26^

 A family of core-shell nanoparticles termed LNCs is engineered to encapsulate drugs. The lipid-based surfactants, lecithin or fatty acids, are in the shell, while the core contains oils like triglycerides.^27^ LNCs typically range in diameter from 20 to 100 nm. The outer hydrophilic surface of LNCs can be modified to ensure that drugs are targeted. Polyethylene glycol (PEG) molecules added to the lipid layer of NCs could, therefore, form a type of lipid-based nanoparticle that will encapsulate the lipid and oily core in a protective layer.^28^ Because LNCs have properties of biological systems, such as viruses, extracellular vesicles, and cell membranes, they are considered biomimetic. The biomimetic nature makes targeting anticancer drugs easier through LNCs.^29^ One property is the inhibition of P-glycoprotein, which contributes to intracellular drug accumulation in cancer cells.

 Negatively charged LNCs circulate for a longer time in the body and are more challenging to be detected by the immune system. LNCs demonstrate the preservation of residency time and further improve mucosal GIT permeability.^30,31^ LNCs can use both passive and active targeting strategies to target cancer. Passive targeting relies on exploiting the characteristics of the tumor, particularly the presence of fenestrated permeable capillaries and the incomplete lymphatic system. The strategy based on active targeting hinges on the mechanism of ligand-receptor binding along with the overexpression of certain markers specific to cancer. The right ligand or biomolecule can be conjugated to NPs, providing the selective accumulation of NPs by specific cancer biomarkers interacting with them at desired sites.^32^ LNCs are promising entities for cancer therapy and can encapsulate a variety of therapeutic chemicals. LNCs might contain immunomodulators, gene therapies, and chemotherapeutics.^33^ The key benefit of integrating various therapeutic modalities in one nanocarrier would be overcoming complex cancer treatment challenges. Given that LNCs optimize the administration and efficacy of cancer treatment while minimizing adverse effects, they indeed make up a promising advancement in oncology.^34^

 This review emphasizes the development of LNCs for drug delivery targeting, especially focusing on successful treatments for various cancers. This review has also emphasized recent research aimed at developing more refined LNC formulations with improved targeting properties and the investigation of new ways to deliver several anticancer drugs.

## Methods for synthesizing LNCs tailored for cancer therapy

###  Phase inversion temperature (PIT) method

**Figure 2:**
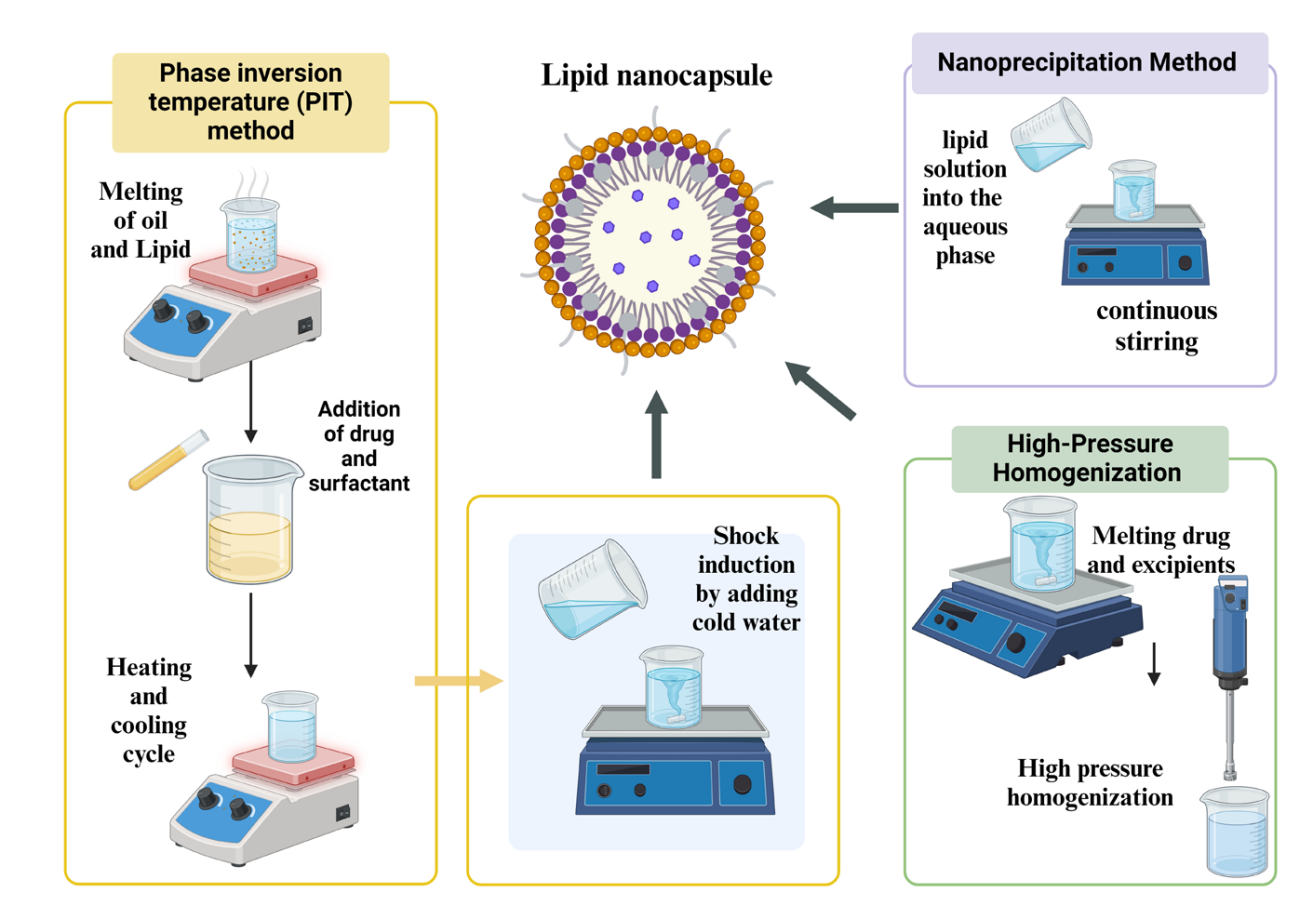
Schematic illustration of the synthesis process for lipid nanocapsules (LNCs)

 In the PIT method, the melting of the oil, lipid, and surfactants in a water bath at 70 °C, medicine is added to the molten mixture. A primary emulsion is prepared by adding salt chloride-infused water. A W/O emulsion is produced by elevating the temperature above the phase inversion in a magnetic stirrer that surrounds the emulsion. An oil-in-water (O/W) emulsion is produced by cooling.^35^ Three heating and cooling cycles are conducted above and below the phase inversion zone. Next, a rapid temperature change is created in the O/W emulsion by adding cold water, which results in an irreversible shock that produces the LNC^36^ as depicted in Figure 2. The sodium chloride is added to maintain the PIT and reduce the formulation’s temperature. Adding cold water instantly to the hot emulsion will produce an irreversible shock that forms LNCs. This procedure helps in the formulation of stable LNCs. After being first presented by Shinoda and Saito in 1969,^37^ the PIT approach is currently extensively employed in industry.^38^ PIT technique is advantageous for preparing LNCs owing to its eco-friendliness, scalability, and the absence of organic solvents. It facilitates efficient encapsulation, reproducibility, and robust physical stability using equipment not equipped with high-shear components, making it favorable for clinical translation over the standard solvent-dependent procedure.^39^ The large-scale production of the LNCs can be formulated using PIT. PIT is the most suitable method for preparing the LNCs, PEGylation of the LNCs, and attachment of active ligands compared to other formulation methods. When the temperature rises, these surfactant types become lipophilic because their hydrogen bonds with water molecules dissolve, and the polyethylene chains dehydrate. High conductivity values and substantial, positive, spontaneous curvature of the surface create O/W emulsions at low temperatures. As the temperature rises, the inherent curvature decreases, and W/O emulsion forms because of a sharp drop in conductivity. Phase-inversion process that transpires in the phase-inversion zone from an O/W to a W/O emulsion.^40^

###  Solvent evaporation method

 The solvent evaporation method is a popular approach for creating LNCs, which works well for encasing hydrophobic anticancer medications. Organic solvent dissolves lipids, oils, and the hydrophobic medication that must be encapsulated. After that, the organic solution is ultrasonically emulsified to create an aqueous phase that contains surfactants or stabilizers. An O/W emulsion is formed when the drug and lipid are dispersed as tiny droplets in the aqueous medium. After swirling the emulsion, the organic solvent is often allowed to evaporate at room temperature or at a lower pressure using a rotary evaporator. LNCs are prepared when the lipid precipitates and the solvent evaporates, resulting in the formation of a solid or semi-solid shell surrounding the drug.^41^ The organic solvent possesses high encapsulation efficiency for a broad spectrum of hydrophobic drugs and bioactive compounds, primarily due to its unique characteristics.^42^

###  Nanoprecipitation method 

 Often, LNCs are produced through a method known as solvent displacement or nanoprecipitation. In an organic solvent, lipids are dissolved and formulated with an aqueous solution containing surfactant to assist in stabilizing the NC that has been produced (Figure 2). The surfactant assists in keeping lipid particles at their nanosized and prevents them from aggregating. The lipid solution is added rapidly to the aqueous phase with continuous stirring. LNCs form upon the diffusion of organic solvent into the aqueous phase during mixing as lipids precipitate out. Uniformly sized LNCs are formed by interacting with surfactant and hydrodynamic shear forces. The remaining solvent is removed through dialysis against water or the evaporation of the organic solvent under low pressure. The purification of the resultant LNCs eliminates any free components or byproducts. The advantages of this method are primarily taken from not making use of high-energy techniques, such as homogenization at high pressure or sonication.^43,44^

###  High-pressure homogenization (HPH)

 LNCs are usually prepared using the HPH process.^45^ This is particularly useful in generating small, monodisperse NCs because it can generate nanoscale particles using high shear forces. Lipids and the drug encapsulated are dissolved or dispersed in a suitable solvent. Such a solution is combined with an aqueous phase in the presence of stabilizers or surfactants to form a coarse emulsion, as shown in Figure 2. The coarse emulsion is usually prepared using a low-shear homogenizer or mechanical stirring. Then, it is further passed through a homogenizer using high pressure. By using high pressure to push the emulsion through a small opening, this apparatus creates strong shear forces that fragment the droplets into smaller nanoparticles.^46^ The process may be repeated several times for the desired particle size and uniformity. HPH produces LNCs that are homogeneously small with a narrow size distribution. Scaling-up of the process is easy; it is suitable for high-volume manufacturing in pharmaceutical industries and very effective for encapsulation of anticancer drugs that are hydrophilic and hydrophobic.^47^

## Therapeutic advantages of LNC in anticancer drug delivery systems

###  LNCs in enhancing the bioavailability of anticancer drugs

 Improving the oral bioavailability of poorly soluble drugs appears promising with LNCs. LNCs, with a unique structure of a surfactant shell surrounding a lipid core, provide many advantages in addition to increased bioavailability. Among new flexible drug delivery strategies for increasing bioavailability, LNCs stand out. Unlike anything that naturally occurs in living cells, the lipid shell typically consists of phospholipids and surfactants covering the oily core of these NCs, making it more like the membranes found in living cells. This design is very helpful in encapsulating, protecting, and targeting the administration of most low-solubility or chemically unstable drugs. This is one of the more long-standing challenges of pharmaceutical sciences: how to enhance the bioavailability of medicinal compounds for their adequate circulation through the body and the formation of the desired therapeutic effect.

 The solubility of several drugs is low, and many of them are unstable in the gastrointestinal tract, rapidly cleared, or degraded by the systemic circulation. This way, poor bioavailability leads to LNCs solving many problems by improving drug solubility, protecting the drugs from enzymatic degradation, and ensuring better absorption and targeted administration. LNCs can increase the oral bioavailability of hydrophilic and hydrophobic drugs by controlling the sustained drug release. They can be engineered to release their payload in a regulated manner, thus increasing therapeutic efficacy and reducing adverse effects. Further, by changing the composition of ligands on their surface, LNCs allow for targeted drug delivery, thereby proving especially useful in disease-treating applications such as cancer, in which the activity of drugs must be localized. Alginate-based rosmarinic acid (RM) LNCs for oral administration were prepared by Gad et al to enhance bioavailability. The findings of their pharmacokinetic study indicated that the peak plasma concentrations (C _max_) of RM for LNC were 61.33 ± 8.89%. This shows that LNCs enhance drug bioavailability with excellent stability.^48^ Arrua et al reported that formulating benznidazole loaded LNCs significantly enhances oral bioavailability. It was discovered that benznidazole was protected by LNCs in simulated gastric fluid and that the drug’s sustained release was made possible in simulated intestinal fluid that included pancreatic enzymes. These lipid nanocarriers penetrated mucus better due to their tiny size and nearly neutral surface charge, and formulations with these characteristics demonstrated less chemical interaction with the glycoproteins in stomach mucus. When benznidazole was added to LNCs, the medication’s permeability across the intestinal epithelium was increased tenfold over that of the non-encapsulated drug.^49^ Ashour et al enhanced the oral bioavailability of Tanshinone IIA using LNCs with sustained release. When Tanshinone IIA LNCs were compared to Tanshinone IIA suspension, the *in vivo* pharmacokinetic analysis showed a significant improvement in both the rate and extent of absorption, with an approximately 3.6-fold increase in the AUC value (*P* ≤ 0.01). Tanshinone IIA LNCs demonstrated a significant increase in half-life and mean residence time, indicating their long-circulating characteristics. The LNCs also have long-circulating properties with enhanced bioavailability, which helps in cancer therapy. LNCs are a flexible and effective drug delivery method that improves the solubility, protection against degradation, absorption, and bioavailability of poorly soluble anticancer drugs.^50^

 To enhance the transdermal bioavailability of the asenapine maleate, El-Tokhy et al formulated the oil-based LNCs for treating schizophrenia. Because terpenes interact with the outer layer of skin and cause the stratum corneum to fluidize, disturbing its tight packing, lavender oil’s high terpene content is the leading cause of its notable permeation enhancement effect. The best LNC formulation demonstrated a comparatively small particle size of 66.1 ± 0.87 nm and optimum stability over a six-month storage period. Transdermal administration of Asenapine Maleate was sustained, suggesting that this could lower the frequency of doses, increase bioavailability, and boost patient adherence.^51^

###  Enhanced drug loading of anticancer drugs using LNCs

 One of the most critical steps in drug formulation involves the encapsulation of drugs into LNCs. In achieving successful encapsulation, it places the medicine inside the core of the LNC to provide the intended therapeutic action. The release profile of the drug from NC can be controlled to provide either triggered or sustained release at the desired location. This controlled release can maximize the therapeutic effect directly at the tumor site without causing systemic side effects. LNCs ensure significant protection against numerous physiological barriers, and this protects drugs from degrading while enhancing the therapeutic effect of anticancer medicines.^52^

 This holistic protection stabilizes the medication, guards it against early degradation, and ensures the medication is released at the delivery site. Bioactive compounds, including carotenoids, can be encapsulated using nano-encapsulation techniques inside the carrier. The active ingredient can be stabilized and made more soluble using nanoencapsulation; also, a regulated release rate, extended action period, and increase in bioavailability are possible for that ingredient.^45^ The encapsulation efficiency refers to the amount of medication effectively incorporated into the LNCs relative to the overall amount of medication used during the formulation process. High encapsulation efficiency is preferred because most drugs that are well-encapsulated in NCs minimize any type of waste and provide the best possible therapeutic benefit. It also enhances the cytotoxic activity against cervical cancer cells when encapsulated in biodegradable NCs. The in vitro experiments revealed that NCs of orlistat showed a more significant cytotoxic effect against the HeLa cell line in comparison to free orlistat in solution and thus proved to be a promising biodegradable delivery system for orlistat as an attractive alternative in the treatment of cervical cancer, especially concerning developing local delivery systems.^53^

###  LNCs for controlled release of anticancer drugs

**Figure 3:**
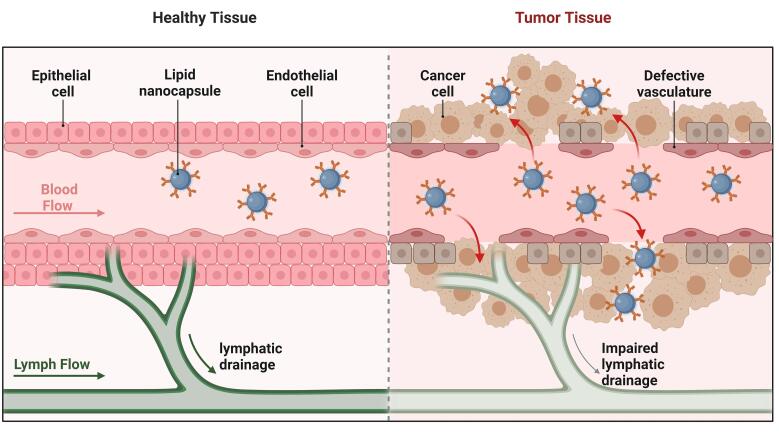
Schematic representation of passive targeting of lipid nanocapsules (LNCs) for the controlled release of anticancer drugs in tumor tissues

 Through various modes of action, LNCs can improve the therapeutic impact of anticancer medication. In addition to enhancing drug solubility and facilitating targeted distribution to cancer cells, they can prevent the encapsulated medication from degrading too soon. By attaching ligands or antibodies to the surface of the NCs that bind selectively to cancer cell markers, active targeting can be provided for targeted delivery instead of passive targeting, which uses the EPR effect. The LNCs, with their high encapsulation efficiency, provide the controlled release delivery of anticancer drugs (Figure 3). Valsalakumari et al highlighted the possibility of using LNCs loaded with paclitaxel (PTX) to treat breast cancer with combinations of several medications that target cancer cells, cancer stem cells (CSCs), and the surrounding microenvironment. The controlled release of anti-cancer drugs enhances the efficacy of the cancer therapy. Hydrophilic polymer surface coating of nanomedicines is a frequently employed modification that prolongs blood residence. The development of new classes of stealth nanocarriers may require the consideration of alternatives to PEG due to the rising prevalence of anti-PEG antibodies in the general human population.^54^ Pautu et al formulated an inventive hydrophilic and pH-responsive copolymer with a lipophilic chain-end that has been used to decorate LNC, giving them the ability to respond to an acidic tumor environment and behave stealthily in physiological settings. Over several weeks, these improved LNCs showed good stability. The pH-responsive LNCs are more effective for targeted and controlled cancer therapy.^55^ Internal stimuli-responsive and enzyme-triggered LNCs can be modified to release the drug in a controlled manner with targeted delivery.^56^ LNCs with controlled release mechanisms can potentially improve treatment outcomes in various medical applications, including cancer therapy, by delivering therapeutic substances more safely and effectively, as shown in Table 1.

**Table 1:** Preparation method, formulation properties, and key findings from in vitro and in vivo investigations of lncs in cancer treatment

**Drug**	**Cancer**	**Preparation method**	**Oils, lipids, and surfactants used**	**Size, Zeta, PDI and %EE**	**Key findings**	**Ref**
Ferulic acid	Colorectal cancer	Nanoprecipitation technique	Labrafac Lipophile WL 1349, Epikuron 145 V, and Tween 80	156.4 ± 0.21 nm -35.8 ± 1.03 mV 0.19 ± 0.02 89.79% ± 0.58	Enhanced anticancer activity over free FA on both HCT-116 and Caco2 cell lines, and apoptosis as the primary mechanism of cell death.	^44^
Itraconazole	Prostate cancer and triple-negative breast cancer	PIT	Labrafac^TM^ lipophile WL 1349, Transcutol HP® and Kolliphor® HS 15	46.0 nm -22.3 mV 0.24 97%	Exhibited greater in vitro cytotoxicity and selectivity towards MCF-7 breast cancer cells.	^52^
PTX	Breast cancer and ovarian cancer	PIT	Labrafac^TM^ lipophile WL 1349, Lipoid® S 75 and Kolliphor® HS 15	120 ± 4 nm -9 mV 0.2 98%	Showed enhanced uptake in MDA-MB-468 cells and indicated that the process of endocytosis is a key factor in PTX-LNCs' toxicity to the cells.	^54^
Hypericin	Skin cancer	PIT	Labrafac® WL 1349, Lipoid® S 100 and Kolliphor® HS 15	76.88 ± 5.95 nm -12.25 ± 2.43 mV 0.19 ± 0.01 89.64 ± 0.44%	Demonstration of exceptional anti-tumour destruction, higher cellular uptake, and improved photocytotoxicity in B16-F10 cells.	^57^
Thymoquinone	Colorectal cancer	PIT	Captex® 8000, lipiod S75® and solutol HS15®	58.3 ± 3.7 nm -3.8 mV 0.04 ± 0.01 87.7 ± 4.9%	Exposed a substantial antitumor growth reduction with no weight loss in mice indicating raised anticancer efficacy.	^58^
Pterostilbene	Hepatic cancer	PIT	Isopropyl myristate, Epikuron 200 and Kolliphor® HS 15	95.06 ± 4.2 nm -8.42 ± 0.56 mV 0.06 ± 0.01 98.02 % ± 0.43	Increased levels of ALT, AST, and AFP were found, reflecting liver damage and advancement towards hepatic carcinoma.	^59^
p722 Ferrocifen	Cutaneous cancers	PIT	Labrafac® WL1349, Lipoid® S75-3 and Kolliphor®HS15	70.0 ± 1.0 nm, -13.5 ± 0.5 mV, 0.15 ± 0.01 and 64.8 ± 13.4	Showed that p722-LNC inhibited tumor growth and enhanced survival in treated mice.	^60^
WS12	Prostate cancer	PIT	Labrafac Lipophile WL 1349, Phospholipon 90G, and Solutol HS15	24.1 ± 0.8 nm -5.1 ± 0.8 mV 0.183 ± 0.007 99%	TRPM8 activation by agonist WS12, when loaded with LNC, greatly inhibited the migration of Prostate Cancer cells.	^61^

ALT, alanine aminotransferase; AST, aspartate aminotransferase; AFP, alpha-fetoprotein; PTX, Paclitaxel; PIT, phase inversion temperature.

## Strategies for functionalizing the surface of LNCs for targeted delivery

 The improved bioavailability and circulation of anticancer drugs are the key criteria for the protection of the encapsulated drug. Surfactant or polymer is incorporated to enhance the stability of the encapsulated drug.^62^ For the delivery of anticancer drugs at the targeted site, while avoiding their gastric degradation, the LNCs are coated with surfactant or polymers. Lipophilic surfactants and phospholipids are comprehensively used as lipid shells.^63^ The amphiphilic nature allows for a stable structure and enhances strong encapsulation of an oily core. The lipid shell further facilitates freeze-drying of the prepared formulation.^64^ It can be converted to a tablet or capsule, which is a patient-compliant drug delivery method.^65,66^ This will stabilize the lipid core by preventing the coalescence of lipid droplets, and the structural integrity of LNC will be maintained. This stabilization is required in maintaining the size as well as the distribution of LNCs in the formulation.^67^

 A lipid shell protects the drug-containing oil from hydrolysis, enzymatic degradation, and other environmental stresses of a biological milieu. It also helps preserve this potency and stability until it eventually reaches its site of action. The lipid shell can facilitate the passage of LNCs across biological membranes, for instance, the blood-brain barrier and the intestinal epithelium, and thus treat brain cancer, avoiding first-pass metabolism.^68^ Because of enhanced permeability, the encapsulated anticancer drug is absorbed faster and has better bioavailability. The lipid shell of LNCs interacts with immune cells, proteins, and cell membranes, as mentioned by Dinh and Yan, 2023.^69^ Surfactants are applied in cancer therapy to protect the lipid and oil core. The nonionic surfactants protect the cancer drugs from the different thermal environments. PEG is used primarily as a nonionic surfactant due to its long-circulating properties. Kolliphor® HS 15, Macrogol (15) - hydroxystearate, Solutol® HS 15, Polyethylene glycol (15) hydroxystearate, polyoxyethylated 12-hydroxystearic acid are the nonionic surfactants which have been used for anti-cancer drug delivery research.

 The Hypericin PEG-LNCs were delivered intradermally with the help of hollow microneedles for skin cancer, which was investigated by Abd-El-Azim et al. The skin barriers during drug delivery were quickly crossed due to the surfactants (Kolliphor® HS 15 and Lipoid® S 100) used to formulate the Hypericin PEG-LNCs. The surfactant plays a significant role during the anticancer drug delivery to the targeted site and in the passive targeting. Surfactants can improve the interface between the LNCs and cell membranes, increasing the cancer cells’ absorption of the drug particles. This may result in an increased concentration of the anticancer medication inside cells. The lipid core and the aqueous surroundings have less surface tension when surfactants are present, which keeps the LNCs from clumping or aggregating. Surfactants can act as a steric barrier to keep the LNCs from becoming too close to one another and preserve their stability over time.^57^ He et almodified LNCs containing peptides with the surfactants to prevent absorption in the intestinal barriers.^70^ This modification of the LNCs protected the drug from gastric environments and crossed the intestinal barriers without leakage of the drug from LNCs. Because of their strong affinity for epithelial cells and *in vitro* stability due to surfactants, LNCs improved intracellularization and transcellular transport. LNC hydrogel coated with Chitosan shows high thermosensitive activity in releasing curcumin to treat oral carcinoma. The coating layer of chitosan helps in a controlled manner and helps to avoid the leaking of the drug from LNCs. The curcumin was distributed throughout the LNCs’ core, and the chitosan coating influenced the curcumin release profile. The Physicochemical properties of the LNCs were not affected by the coating with chitosan.^71^

 PEGylated LNCs are one strategy that enhances circulation time in the systemic circulation and protects encapsulated drugs. The PEGylation shows good gastric stability and avoids degradation of drugs and LNCs in the systemic circulation.^72^ The PEGylated LNCs, or PEG-LNCs, pass through the stomach and enter the small intestine. The PEG covering shields the LNC from enzymatic degradation by the upper gastrointestinal tract and the acidic, hostile environment of the stomach.^73^ PEG-LNCs can penetrate the mucous layer and are absorbed through an endocytosis mechanism by intestinal enterocytes. PEG-LNCs integrate with chylomicrons, lipoproteins formed by the breakdown and intake of dietary fats within the enterocytes.^74^ PEG-LNCs that contained chylomicrons were released into the lacteals, and lymphatic capillaries were found in the villi of the small intestine. The drug carried with it in PEG-LNCs travels and penetrates with chylomicrons into the intestinal lymphatic capillaries.^75^ The chylomicrons carry the PEG-LNCs through the thoracic duct and the mesenteric lymph nodes further into the subclavian vein, and then into the systemic circulation.^76,77^

 The surface modification of LNCs must be established so that these drug delivery systems can be given stealth characteristics and improve the uptake into the tumor microenvironment. The synthesis of pH-responsive and other amphiphilic poly(N-vinyl amide)-based polymers, as well as their use as LNC modifiers to enhance drug delivery systems.^78^ To prevent the intestinal drug absorption of the anticancer drug in the colon, Ramzy et al, coated anisamide polymethacrylate shells on LNCs. With the use of anisamide as a ligand for sigma receptors, which are generally overexpressed by cells involved in colon cancer, and Eudragit S100 as a pH-sensitive polymer, conjugated as well as non-conjugated NC loaded with thymoquinone were produced to target colon cancer. The NC conjugated with anisamide was cytotoxic to HT-29 cells compared to free thymoquinone and the non-conjugated NCs. This can be explained by the overproduction of sigma receptors of HT-29 cells, which leads to an enhancement of NC uptake.^79^ LNC functionalized with gold-III and bevacizumab showed cellular internalization that was reliant on the C6 cell culture, as demonstrated by de Cristo Soares Alves et al. The Chick Embryo Chorioallantoic Membrane assay demonstrated a 5.6-fold reduction in bevacizumab dose when LNC was functionalized with gold-III, and bevacizumab was compared to bevacizumab solution, indicating a greater antiangiogenic efficacy of the compound.^80^

## Co-delivery system using LNCs

 Bridging the structural benefits of polymeric nanoparticles and liposomes, LNCs consist of a lipid core coated with a surfactant shell that allows the loading of various therapeutic agents. LNC-based co-delivery strategies provide synergistic effects, better pharmacokinetics, and enhanced tumor targeting by the EPR effect. Additionally, LNCs can shield drugs against degradation, promote controlled release, and be functionalized for active targeting. These qualities render LNCs a viable platform for conquering multidrug resistance and enhancing the effectiveness of cancer treatments.^81,82^ Basu et al investigated the co-delivery of PTX and salinomycin (SAL) in LNCs, which improved efficiency against breast cancer and stem cells. This work reveals the therapeutic promise of LNCs co-loaded with PTX and SAL as a targeted treatment for breast cancer. The co-loaded dual-drug LNCs targeted efficiently both CSCs and breast cancer cells (MCF-7), which showed greater cytotoxicity and apoptosis induction than single-drug loaded formulations. Increased internalization, synergistic killing of cells, and mammosphere inhibition highlight the drug-resistant cancer therapy potential of PTX-SAL co-loaded LNCs to improve the outcome of breast cancer treatments. The formulation showed high encapsulation efficiency, sustained drug release, and efficient cellular uptake. These results indicate that co-encapsulation of chemotherapeutic and anti-CSC agents in LNCs presents a potent strategy to tackle tumor heterogeneity, reduce recurrence, and enhance overall breast cancer therapy.^83^ Ashour et al investigated lactoferrin-coated flaxosules for efficient piperine and flaxseed oil co-delivery in breast cancer treatment. The research proved that LF-PIP-FLX-LNC (lactoferrin-coated piperine and flaxseed oil-loaded LNCs) showed significant anticancer activity compared to free piperine solution. Triple-negative MDA-MB-231 cells provided in vitro data with a ~21-fold decrease in IC50 and a 2.5-fold inhibition in wound closure, which proves potent cytotoxicity and antimigration action. Tumor volume was reduced by 40-fold with LF-PIP-FLX-LNC treatment in an Ehrlich ascites breast cancer mouse model. LF-PIP-FLX-LNC induced its antitumor activities by modulating apoptosis and autophagy via the AMPK/mTOR pathway. These observations place LF-PIP-FLX-LNC as a promising nanoformulation of phytotherapy for targeted breast cancer treatment.^84^ The co-delivery of anticancer drugs in LNCs presents a powerful approach to further improving cancer treatment. LNCs offer the following benefits such as slow release of drugs, improved drug encapsulation, and better targeting of bulk tumor cells and CSCs, which typically cause therapeutic recalcitrance and relapse. The simultaneous co-encapsulation of dual drugs enables a synergistic impact, enhancing treatment efficacy while avoiding side effects. In addition, the controlled release profiles and stability of LNCs facilitate effective drug delivery to tumor sites, minimizing systemic toxicity. LNCs are a promising platform for overcoming the difficulties of cancer treatment, providing a potential solution to enhance the efficacy and specificity of chemotherapeutic approaches.

## Cellular uptake of LNCs by endocytosis pathways

 Cells internalize the LNCs through the basic process of endocytosis. Optimizing the distribution and therapeutic efficacy of LNCs in cancer treatment requires a thorough understanding of the unique endocytosis pathways that cancer cells use to internalize LNCs.^85^ The four primary endocytosis pathways are clathrin-mediated, caveolae-mediated, micropinocytosis, and phagocytosis. It is conceivable to increase targeted drug delivery, get around resistance mechanisms, and improve overall treatment outcomes by customizing LNC design to take advantage of endocytic pathways.^86^ A study using an *in vitro* coculture model to assess the absorption of various LNCs in the intestine was conducted by Kaeokhamloed et al. This study showed that molecular drugs are absorbed mainly by the passive route, controlled by the chemical rather than the biological characteristics of the membrane. The LNCs, however, are taken up via active transport in the Caco-2 model. The Kolliphor® HS-15 on the outer shell of LNCs may directly interact with the lipid-rich microdomain on the cell surface, leading to endocytosis. The addition of the human microvascular endothelial cell type 1 layer in the coculture model was thought to be the cause of the variation in LNC absorption between this and the Caco-2 models because it might influence the biological absorption process of the cell but not the chemical properties of the membrane, which could be the basis for molecular drug absorption.^87^

 A biological cell line study of LNCs uptake in human breast adenocarcinoma line (MCF-7) was estimated by Vasconcelos et al, LNCs enhanced the stability of lycopene and increased its toxicity against MCF-7 cancer cells, prevented intracellular growth enzyme production in human microglial cells, and did not compromise erythrocyte membrane integrity, highlighting its potential as a cancer treatment. Since nanoparticles are inevitably broken down and destroyed by acids and enzymes, the endosome pathway is regarded as a degradative intracellular transport system, which can harm the effects of encapsulated medications.^88^ The proton sponge effect of excellent pH buffering, osmotic lysis brought on by pH-responsive nanoparticle disassembly, swelling effect of pH-responsive nanoparticles, and membrane destabilization brought on by pore formation and photochemical internalization are examples of sophisticated strategies used to promote endosomal escape of nano drugs.^89^ LNCs are more successful for targeted drug delivery in cancer therapy because they comprehend and take advantage of these endocytosis mechanisms, which will enhance therapeutic outcomes and minimize adverse effects.

## Biodistribution and pharmacokinetics of LNC

 The reticuloendothelial system (RES) often sequesters LNCs, mainly accumulating them in the liver and spleen because of the high phagocytic activity demonstrated by the macrophages within those organs. Formulations of LNCs, prepared with the appropriate targeting ligands, can accumulate within tumors through mechanisms of active targeting and enhanced permeability and retention (EPR) effect. Depending on how their surface is modified and the size of LNCs, they can also spread to the kidneys, lungs, and other tissues.^90^ Several variables, like surface charge, particle size, and circulation time, are involved in the biodistribution of LNCs. The PEG coating can prolong circulation duration and reduce RES uptake.^91^ LNCs whose diameter is less than 100 nm typically have longer tissue penetration times and periods of circulation. Targeting LNCs to tissues or cell types can be achieved through the attachment of antibodies, peptides, or other molecules to them.^92^

 The biodistribution, cellular absorption, and excretion of LNCs from the body are considerably influenced by size and surface charge. Colloidal nanoparticles can be directed to tumor tissue preferentially by its leaky vasculature, known as the EPR effect. The dimensions, 10-200 nm, and surface charge, often referred to as zeta potential, determine the degree to which well-suspended NCs maintain suspension and engage with biological membranes.^93^ Positively charged LNCs are susceptible to being cleared early by the mononuclear phagocyte system (MPS), considering their electrostatic interactions with negatively charged cell membranes; however, they can improve cellular absorption.^94^ Stability refers to the ability of LNCs to preserve their size, surface charge, and drug content encapsulation over time. This depends on temperature, the nature of lipids, and the stabilizing agents. This requires NCs for their shelf-life to be extended indefinitely with no signs of degradation and aggregation so that their therapeutic activity can be maintained.^95^ LNCs can be administered through intravenous, oral, and topical routes. The route of administration hugely influences the extent of absorption and the pharmacological activity of LNCs. Once administered, LNCs are distributed throughout the body.

 In IV delivery, the distribution rate and the extent are governed by the administered dose and the physicochemical properties of the LNCs. LNCs are metabolized primarily by the liver. The components of the LNCs and the encapsulated medicines come out as urine and feces from the digestive tract due to excretion by the kidneys. The LNCs release the encapsulated drug through the action of the enzymes in the liver, dissolving the lipid portion of the LNCs. Composition, surface properties, and size impact the excretion profile of the LNCs.^96^ LNCs’ half-life in circulation is one of the significant pharmacokinetic parameters.^97^ PEGylation and other surface modifiers that decrease renal clearance and RES uptake extend the half-life.^98^ In a comparison of LNCs and suspension of PTX and Curcumin, the absorption characteristics (C max and AUC0-t) were revealed to be significantly different, as reported by Rahman et al (*P* < 0.05). The C max and AUC0-t of PTX resulting from the LNC were nearly greater than the free drug suspension. The C max and AUC0-t of the curcumin-loaded LNC were far more significant (*P* < 0.05) than those of the PTX and Curcumin suspension.^99^ The lipophilic nature and nanosized size of the LNC might allow for the uptake of nanocarriers through paracellular and intercellular channels, thus fully justifying the significant increase in drug absorption metrics. For PTX, TPGS-LNCs had increased pharmacokinetic parameters, such as AUC(0-α) and t1/2, with respect to the commercial formulation. PTX-loaded TPGS-LNCs showed an increase in AUC(0-α), where a t1/2 increase to approximately 3.18 times was reported with respect to the commercial product. Most probably, the sustained release characteristics of TPGS-LNCs and the extended circulation duration for PTX are the reasons for the enhanced pharmacokinetic characteristics of PTX and curcumin.^100^ The loading of the drugs increased the pharmacokinetic activity of the LNCs compared to the pure drugs, proving the enhanced pharmacokinetic properties of LNCs in anticancer drug delivery.

## Applications

###  LNCs in reducing systemic toxicity and targeted delivery to tumor cells

**Figure 4:**
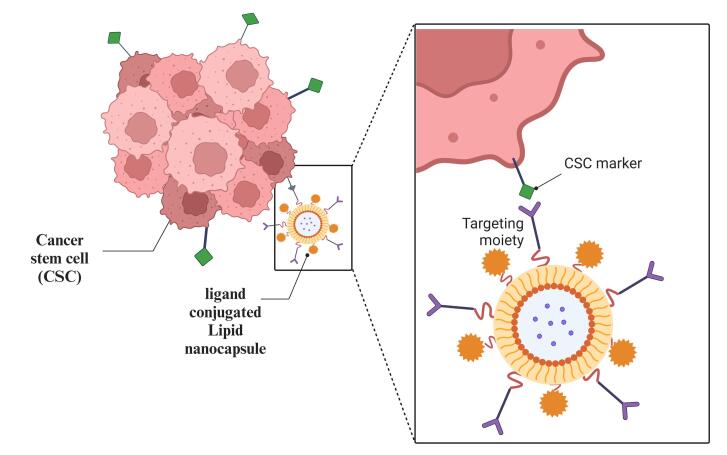
Schematic illustration of ligand-conjugated lipid nanocapsules enabling active targeting

 The focused drug delivery in which the LNPs are targeted to the tumor site provides more effective treatment and improved bioavailability. LNCs can be used for active and passive targeting by altering the surface. Adding a targeting ligand to LNCs is simple and efficient for targeted delivery, as shown in Figure 4 and Table 2. Pinton et al worked on LNCs, which were altered to influence their absorption by leukocytes and tumor-infiltrating cells circulating in the blood. Pinton et al made use of these characteristics to tailor LNC to target immunosuppressive populations in a way that minimizes T cell internalization. The size of the LNCs prepared by Pinton et al, was 100 nm with a positive charge on the surface. This strategy can be adapted to other tumors and develop novel, individually customized, targeted immunotherapy-based drug delivery systems.^101^ The neutral and positively charged particles are more effective in targeting the LNCs for blood and tumor sites.^102^

**Table 2:** Summary of ligands and target receptors, and associated cancer types for surface functionalization of lipid nanocapsules

**Drugs**	**Cancer Therapy**	**Ligand**	**Targeted Receptors**	**In Vitro Outcomes of the Study**	**Ref**
Thymoquinone	Colon tumors	Anisamide	Sigma-1 receptor	HT-29 cells overexpressing sigma receptors were more susceptible to the cytotoxic effects of anisamide-targeted TQ LNCs than those of non-targeted TQ LNCs.	^79^
Doxorubicin	Ovarian and bladder tumor	Folic acid	Folate receptor	The increased absorption capacity of FA-conjugated LNCs and increased rate of induced cell death in the bladder (T24) and ovarian (OVCAR-3) lines.	^103^
Hyaluronic acid	Tumor-associated macrophages	Monosaccharide mannose	Mannose receptor	HA combined with mannose resulted in an enhanced biodistribution with strong infiltration of tumor-associated macrophages and increased accumulation in the tumor, as well as a notable increase in NCs absorption by M2 macrophages.	^104^
Docetaxel	Lung tumors	tLyp1 peptide	Neuropilin-1 receptors	Efficiently interacted with Neuropilin-1 receptors, overexpressed in A549 lung cancer cell cultures, enabling dual-targeted delivery and improved cellular uptake.	^105^
Docetaxel	Lung tumors	Anti-Tn antigen	Lectin receptors	Anti-Tn antigen increases LNC internalization and reduces cell viability more effectively by internalizing the cytoplasm of the cell.	^106^
Curcumin	Brain tumors	CD44 binding peptide	CD44 standard isoform	Curcumin fluorescence intensity is higher in CD44-LNCs as compared to cell numbers, and peptide-functionalized NCs accumulated 51.5% more in cells that overexpress CD44.	^107^
Hymecromone	Lung tumors	Hyaluronic and Folic acids	CD44 and folate receptor	Potentiality showed a tumour growth suppression via apoptotic mechanisms and angiogenesis inhibition in a urethane-induced lung cancer model, along with an excellent safety profile	^108^

LNC, Lipid nanocapsule; FA, Folic acid; HA, Hyaluronic acid.

 The prolonged release of drug particles from polymer hybrids was responsible for the strong anticancer action. This release could enable the entrapped medication to be delivered to tumor tissues at sufficient concentrations after systemic treatment.^109^ Functionalization of several polymer systems has been achieved using covalent and non-covalent conjugations. Targets for the surface functionalization of nanoparticles are frequently specific peptides and antibodies.^110^ Because folate receptors are severely restricted in most normal tissues but broadly expressed in certain tumor cell lines, folic acid (FA) has been employed as a ligand for the selective targeting and delivery of medicines in tumor cells.^111^ During cancer conditions, the expression of the folate receptors helps the folic acid find the targeted site. Active targeting can be achieved by conjugating targeted ligands like FA. FA-conjugated Doxorubicin LNCs are formulated by Cé et al, to target the ovarian and bladder cancer cells. In this research, the FA was easily conjugated to LNCs, forming FA-conjugated LNCs. When organs, tissues, cells, or subcellular domains are sick, ligands are chosen to bind surface molecules or receptors that are overexpressed.^103^ Targeting the folate receptors has garnered significant attention in developing anti-tumor drugs, including DNA.^112^ Due to the disulfide bonds in the NC shell, the FA-LNCs demonstrated good reduction-responsive release properties for hydrophobic anti-cancer medications. FA-LNCs have a controlled drug delivery system for targeted distribution and triggered release of hydrophobic drugs in the biomedical field.^113^

 Grolez et al used LNCs containing a Transient Receptor Potential Melastatin 8 channel (TRPM8) agonist to block prostate cancer cell motility by activating a channel. The TRPM8 partially inhibits prostate cancer cell migration. LNC-encapsulated WS12 enables the use of lower agonist doses for TRPM8 activation. WS12 targets cancer cell migration by acting as an agonist for TRPM8 activity and encasing it in LNCs. The research investigates how the encapsulated WS12 affects the migration of prostate cancer cells mediated by TRPM8 both *in vivo* and *in vitro*, and demonstrates WS12’s affinity and specificity for TRPM8 by making it a successful channel agonist. The potential therapeutic applications of WS12-loaded NCs have the feasibility of functionalizing the LNCs for anticancer medication delivery on a targeted basis by activating channels.^61^ The development of an implantable therapeutic hydrogel by Gazaille et al, promises the continuity of treatment for patients with glioblastoma between chemotherapy and surgery. The neurofilament subunit (NFL)-tubulin binding site (TBS) amino acid 40-63 peptide was used to formulate the LNC-based hydrogels. Without altering the mechanical characteristics of the hydrogel, the peptide was completely and instantly adsorbed to the LNC surface, where it stayed until the hydrogel disintegrated. Research conducted *in vitro* on Glioblastoma cell lines showed that the presence of NFL accelerated the LNC’s internalization and increased its cytotoxicity. The gemcitabine-loaded LNC with adsorbed NFL-TBS.40-63 peptide may target the non-resected Glioblastoma cells and greatly delay or even suppress the appearance of recurrences, as demonstrated by the final *in vivo* investigations conducted in the murine Glioblastoma resection model.^114^

 Hyaluronic acid (HA) targets lymphatic vessel endothelial hyaluronan receptor (LYVE 1), expressing lymphatic endothelial cells. Vesicular endothelial growth factor (VEGF) is responsible for cell proliferation, which also helps in the overgrowth of tumor cells during malignant conditions. Janik-Hazuka et al, conjugated HA for garlic oil loaded NCs.^115^ This study showed LNCs hold great potential as carriers for tackling major issues such as hydrophobic ingredient transport, unwanted interactions between those compounds and bodily fluid components, and chemical instability at low pH or oxygen conditions. Garlic oil, having anticancer activity, possesses bioactive molecules such as allicin that trigger apoptosis and suppress the growth of cancer cells. Targeted delivery to cancer cells, especially CD44 receptor-overexpressing cancer cells, is realized by encapsulating garlic oil in HA-conjugated LNCs, making it more effective and lowering off-target toxicity. The hydrophobic character of garlic oil is overcome using LNC, enhancing its stability and bioavailability. Also, its antioxidant and anti-inflammatory properties add to its potential in inhibiting tumor growth and improving chemotherapy effectiveness, making it a promising agent in cancer treatment. Based on Janik-Hazuka et al, HA-NCs could serve as flexible delivery vehicles that enhance patient safety. The long-term stability highlights the finding that HA-based systems are stable regardless of the core material.^115^ LNCs exhibit several positive characteristics, including the absence of aggregations with serum components, a decreased hemolysis effect, and stability in the presence of the primary digestive system enzymes and low pH levels, all pointing to the extensive therapeutic applications. Mariño et al modified HA-NCs for targeting tumor-associated macrophages.^104^ In solid tumors, the mannose-functionalized HA NCs were able to reach TAMs. Following intravenous delivery, there was a discernible improvement in the biodistribution profile of the HA-NCs, with greater blood circulation time, decreased hepatic absorption, and a notable rise in tumor accumulation. These findings offer a range of adaptable HA-NCs for macrophage targeting in solid tumors.

 Peptides such as cell-penetrating peptides (e.g., TAT), tumor-homing peptides (e.g., RGD), and enzyme-responsive peptides can be anchored onto the LNC surface to facilitate selective uptake by tumor cells, enhance penetration into solid tumors, and facilitate stimuli-responsive drug release. Their ease of synthesis, small size, and high specificity render them valuable candidates for active targeting strategies.^70^ In this research conducted by Kim et al, a PEGylated polypeptide-derived lipid nanocapsule (PLN) was developed to improve erlotinib delivery for treating non-small cell lung cancer. The PLN had a core-shell morphology with 200 nm particle size and -20 mV surface charge. The PEGylated polypeptide shell acted as a molecular barrier for sustained release, and poly (L-aspartic acid) ensured pH-responsive drug release in the acidic tumor microenvironment. ERL-loaded PLNs exhibited increased cytotoxicity in lung cancer cell lines and strongly enhanced tumor regression in a xenograft model. Tumor volumes were reduced by 2-fold compared with free erlotinib upon PLN treatment, indicating its potential as a potent, targeted cancer drug delivery system.^72^ The study by Zhang et al presents the construction of a pH-sensitive, PEG-shedding lipid nanocapsule system (PEG-S-LNCs) for improved delivery of disulfiram (DSF) in copper-augmented cancer treatment. The nanocarriers were modified for cell entry using TAT peptide (TATp) and PEG coatings responsive to acidic tumor microenvironments. The findings confirm the nanocarriers’ potential to utilize tumor acidity for PEG trigger-induced removal, TATp-mediated uptake improvement, and copper-based cytotoxicity. TATp-DSF-S-LNCs present a promising approach to targeted, environment-sensitive cancer therapy with enhanced bioavailability and therapeutic activity. TATp-LNCs enable the internalization of nanocapsules into cancer cells by energy-independent translocation or endocytosis, significantly enhancing drug delivery efficiency. TATp-LNCs help translocate DSF-loaded LNC across cellular membranes, enabling efficient cytoplasmic release of drugs, which is essential for copper-mediated anticancer activity.^116^ This research by Wang et al explores the synthesis of RGD modified LNCs for improved targeted delivery of curcumin (Cur) to cancer cells. Chitosan, biocompatible and biodegradable, encapsulated Cur, enhancing its controlled release and stability. NCs were developed to exploit the EPR effect. RGD peptides helped in tumor-specific targeting by interacting with the αvβ3 integrin, which is typically overexpressed in some tumors. In vitro experiments using MDA-MB-231 breast cancer cells proved that RGD-modified LNCs greatly enhanced cellular uptake and cytotoxicity compared with free Cur, increasing cancer cell death due to extended drug release and improved internalization. These findings indicate that RGD-modified LNCs have tremendous potential for targeted cancer therapy, although more in vivo experiments are required to confirm their therapeutic effects.^117^ However, monoclonal antibodies or antibody fragments impart high-affinity and antigen-specific binding to allow LNCs to selectively bind and internalize into tumor cells that display surface markers (e.g., HER2, EGFR). This improves the therapeutic index of encapsulated drugs as well as minimizing systemic toxicity.^118^ The research by Teijeiro-Valino et al introduces a dual-functional drug nanocarrier, hybridizing HA nanocapsules with the tumor-targeting peptide tLyp1, for improved targeting and penetration in cancer treatment. The nanocapsules have specific interaction with CD44 and NRP1 receptors, which are overexpressed in A549 lung cancer cells, exhibiting dual targeting capabilities to the tumor and lymphatics. In a 3D co-culture in vitro model, the nanocapsules exhibited efficient binding with NRP1 receptors. In vivo, HA-tLyp1 nanocapsules loaded with docetaxel had a remarkable 37-fold increase in tumor accumulation over Taxotere. This increased accumulation resulted in substantial tumor growth regression and decreased metastasis in an A549 orthotopic lung cancer model. The nanocapsules also had similar efficacy to a tenfold greater dose of Abraxane in a pancreatic patient-derived tumor model. These results emphasize the significance of multifunctionality in enhancing the therapeutic effectiveness of cancer therapies, especially to target primary tumors and metastasis.^105^

###  Synergistic effect of LNCs for anticancer drug delivery

 A common challenge in cancer treatment is getting beyond the complex resistance mechanisms that cancer cells build up against therapeutic medicines. Using numerous therapeutic compounds in combination to boost efficacy and overcome drug resistance is known as the synergistic effect bypass (Table 3), and it is one promising approach to addressing cancer drug delivery.^119,120^ This strategy uses how various medications interact with one another to create a combined impact stronger than the sum of their parts. In cancer treatment, the synergistic effect bypass strategy is frequently employed, and novel combinations of chemotherapy, targeted therapy, immunotherapies, and radiation therapy are investigated.^121^ LNCs help target multiple pathways and overcome drug resistance, which promotes the synergistic effect.^122^ Tsakiris et al worked to treat various tumors with an intravenous combination therapy that uses LNCs as a medication delivery vehicle.^123^

**Table 3:** Surface modification strategies of lipid nanocapsules for combinational cancer therapeutics

**Drugs**	**Cancer therapy**	**Surface modification**	**In vitro outcomes of the study**	**Ref**
Rolapitant and deferasirox	Breast cancer	PEGylation	Strong cytotoxic action against MCF7 cancer cells, with each medication having a better antitumor impact.	^73^
Docetaxel and thymoquinone	Breast cancer	D-α-tocopheryl polyethylene glycol 1000 succinate and ethylene glycol 2000	Enhanced cytotoxicity with anti-metastatic, anti-apoptotic, and protein binding resistance that validates prolonged blood circulation.	^124^
Docetaxel and thymoquinone	Breast cancer	Chitosan grafted LNCs	Increased intracellular delivery of the dual payload, notably enhanced cell cytotoxicity in MCF 7 cells, and controlled release of the payloads.	^125^
Docetaxel and quercetin	Prostate cancer	Poly-lactide-co-glycolide modified with PEGylation	Luteinizing hormone-releasing hormone conferred more cellular absorption and increased tumor accumulation capabilities, whereas PEG gave the NC a stealth coating by stabilizing serum.	^126^

PEG, Polyethylene glycol; LNC, Lipid nanocapsule.

 SN38 is primarily metabolized in the liver, reducing oral bioavailability. To overcome this, the LNCs were formulated, which showed the enhanced inhibition of tumors. The combination of the drugs showed a synergic effect with good efficacy. The in vitro investigations demonstrated that co-encapsulation in LNCs significantly reduced hemolysis while demonstrating no discernible difference in the cytotoxicity of free and encapsulated medicines. Ultimately, the in vivo trial demonstrated that the combination of SN38 and regorafenib-LNCs reduces the growth of colorectal cancer and lengthens the median survival period. The formulation showed high drug loading capacity with enhanced targeting of six drug combinations to collateral cancer. This investigation suggests that the LNCs are efficient for combination cancer therapy in both oral and IV routes. Radwan et al developed LNCs to examine the *in vitro* synergistic therapeutic impact of a new LNC-encapsulated combination of capsaicin and 5-fluorouracil.^127^ Compared to the cytotoxic effect of each drug-loaded LNC given alone, the *in vitro* biological investigations showed encouraging results regarding the synergistic effect of the combined drug. These investigations revealed that LNCs were highly applied in cancer therapy for synergistic effects. Treatment for resistant breast cancer may be improved with the use of well-thought-out combination nano-therapy techniques. The combination of docetaxel and thymoquinone co-encapsulated within long-circulating PEG-LNCs is reported by Zafar et al,^124^ LNCs provide an efficient method for successful combination chemotherapy with fewer unintended effects.

 Due to several drawbacks, traditional cancer treatment methods like chemotherapy and radiation must be combined with various complementary modalities to increase their effectiveness. Radiation therapy (radiosensitizer) and chemotherapy (chemosensitizer) may work in concert with hyperthermia as an adjuvant therapeutic approach for cancer. Traditional hyperthermia techniques have poor selectivity and impact both healthy and tumorous tissues. A temperature gradient is also produced in the tissues along the heat source’s path; this gradient is particularly severe in the case of deep-seated malignancies. By localizing around or within tumoral tissue and causing localized hyperthermia, LNC-induced hyperthermia can overcome these disadvantages. Thus, the focus of this study was the principles of NC-induced hyperthermia and, more crucially, its synergistic effects with conventional chemotherapy or radiation therapy. In cancer therapy, heat-producing nanostructures like gold nanoparticles, iron oxide nanoclusters, and carbon nanoclusters are highlighted together with non-heat-producing nanostructures, including lipid-based, polymeric, and silica-based NCs that act as carriers for heat-producing LNCs.

###  Delivery of nucleic acids 

 The ability of LNCs to interact with cell membranes is facilitated by their lipid makeup, which permits effective endocytosis and the consequent release of nucleic acids into the cytoplasm. LNCs can also be functionalized with ligands for targeted distribution to tissues or cell types to improve treatment outcomes and lessen off-target effects (Table 4). Because of their biocompatibility and modifiable surface characteristics, LNCs have been investigated in several therapeutic domains, including cancer immunotherapy, gene therapy, and vaccine development. Cancer cells can be developed to produce autonomous cell death by introducing prodrugs and the class of cytotoxic and tumor-suppressive protein genes.^112^ LNCs can encapsulate small interfering RNA, thus silencing the oncogenes critically involved in cancer development. For instance, LNCs may be used to deliver siRNA that targets KRAS, a gene commonly mutated in malignancies, thereby inhibiting tumor formation. Several issues exist about the delivery of siRNA. For instance, poor stability, limited circulation in the bloodstream, growth of tumors, and inefficient intracellular release of siRNA severely limit siRNA-based therapy.^128^ Huang et al explored Cation-free siRNA-cored NC for tumor-targeted RNAi therapy. The survival rate of PC-3 tumor-bearing mice was highly elevated by NCs incorporating siRNA targeting polo-like kinase 1, which also highly suppressed the tumor growth in the absence of side effects from cation-associated toxicity.^129^

**Table 4:** Summary of gene delivery systems using lipid nanocapsules

**Gene system**	**Target cells**	**Functionalization**	**Results**	**Ref**
siRNA-PLK1	PC-3 prostate cancer cells	cRGD ligand	Effective siRNA delivery, tumor-specific gene silencing, inhibited tumor growth, and no cation-related toxicity.	^129^
siRNA (DOTAP/DOPE lipoplex)	SK-Mel28 melanoma cells	PEGylation	High encapsulation efficiency, no cytotoxicity at concentrations up to 500 ng/mL, efficient gene silencing, appropriate for passive tumor targeting.	^130^
Bcl-2 siRNA	Metastatic melanoma cells	PEGylation	Accomplished effective Bcl-2 knockdown and increased cytotoxicity, leading to a reduction of about 50% in tumor size.	^131^

###  Lymphatic delivery 

**Figure 5:**
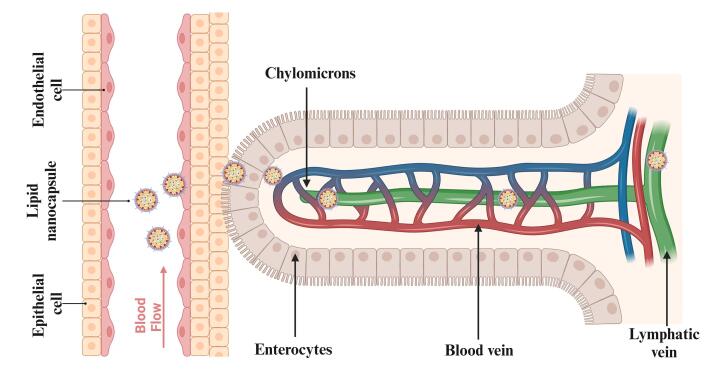
Mechanism of lipid nanocapsule absorption via the lymphatic system

 The lymphatic delivery of the anticancer drugs through the subcutaneous route by the LNCs shows greater efficacy. PEG improves the lymphatic uptake in the endothelial cells and reduces cellular uptake, as shown in Figure 5. Surface PEG density also greatly affects nanoparticle uptake by the lymphatic system. When the density of PEG is low, nanoparticles become less “stealthy” and tend to have higher interactions with lymphatic endothelial cells or macrophages in lymph nodes, thus causing enhanced lymphatic uptake. But it also leads to faster clearance from circulation through recognition by the immune system. Conversely, higher PEG densities offer a more pronounced stealth effect, decreasing immune detection and prolonging circulation time, yet this can inhibit nanoparticle uptake via the lymphatic system. There is generally a preferred PEG density range that balances reduced immune recognition and efficient lymphatic uptake. This balance is essential for optimal exploitation of the therapeutic potential of nanoparticles, enabling targeted delivery to lymphatic tissue while ensuring extended circulation for subsequent accumulation at cancer sites. Thus, meticulous modulation of PEG density is critical for ensuring optimal biodistribution, enabling effective lymphatic system targeting, and avoiding premature immune clearance. The PRG also helps control the release of drug molecules at the tumor site. Increasing PEG density on the surface of nanoparticles can improve and optimize transport across lymphatic endothelial barriers.^132^ A coating of amphiphilic lipids, made up of phospholipids, envelops a core of neutral triglycerides that make up chylomicrons.

 Lipid nanocapsules were observed in the brush border of microvilli, enterocytes’ apical cytoplasm, smooth endoplasmic reticulum, and secretory vesicles. LNPs were found in large quantities in the intestinal villi’s lamina propria, where they were accumulated and permeated.^133^ After subcutaneous injection, Abellan-Pose et al investigated the impact of particle sizes ranging from 100 to 200 nm on the biodistribution of polyglutamic acid-PEG NC. The findings demonstrated that compared to NC with a size of 200 nm, those with a size of 100 nm accumulated in the lymph nodes more quickly.^77^ With the use of gel technology based on LNCs loaded with a lipophilic pro-drug of gemcitabine injected subcutaneously, which is investigated by Wauthoz et al, it was found to be possible to combat the metastases locally and target adjacent lymph nodes, including the mediastinal lymph nodes, by showing a survival increase comparable to that of an intravenous treatment that prevents the primary tumor from spreading the metastases.^134^ PEGylation increased in circulation time, as PEG particles showed less uptake into the liver than non-PEG particles. The intestinal lymphatic system takes up PEG molecules via transintestinal lymphatic transport. This entails internalizing PRG molecules from the intestinal lumen into intestinal epithelial cells and further to lymphatic vessels within the intestinal villi. This lymphatic delivery of the LNCs with the help of PEG will avoid first-pass metabolism and enhance the drug bioavailability. The anticancer drugs generally possess low solubility and lower bioavailability, which is bypassed by the lymphatic delivery of the LNCs.^135,136^

## Limitations and toxicity of LNCs

 Despite their promising therapeutic potential, LNCs have several limitations that need to be seriously tackled before clinical translation. One of the most significant concerns is immune recognition and clearance, especially with PEGylated LNCs, as repeated doses may trigger anti-PEG antibody formation, causing rapid blood clearance and decreased efficacy. Furthermore, instability in biological environments could lead to premature leakage or aggregation of drugs, hindering targeted delivery.^137^ Although PEGylation has been used for a long time to enhance the pharmacokinetics and blood circulation time of LNCs, recent reports have shown profound immunological disadvantages. One of the main issues is the phenomenon of accelerated blood clearance (ABC), where the initial injection of PEGylated nanocarriers triggers anti-PEG antibodies, promoting quick clearance of the following doses, lowering the therapeutic effect. In addition, complement activation-related pseudo-allergy (CARPA), an immunological hypersensitivity reaction due to activation of the complement system, has been seen with PEGylated products. The inflammatory response can cause severe side effects and restrict the clinical safety of these nanocarriers.^138,139^ The increasing awareness of these immunogenic issues has led regulatory bodies like the U.S. FDA to require the assessment of anti-PEG antibody responses for new PEG-containing therapeutics. HA-functionalized LNCs had higher cytotoxicity against squamous cell carcinoma and better immunostimulant and apoptosis activity in lower doses than free IMQ and commercial preparations. The paper presents significant limitations. These are a small sample size, few variables that were measured, and an abbreviated evaluation period, which may limit the external validity of the results. Additionally, although the targeting efficacy in the observed HA-CD44 interaction is encouraging, long-term safety and robustness in widely differing populations of patients have not been validated. Future research must include larger preclinical investigations, increased variable sets, and extended treatment time courses to assess extended efficacy and safety.^140^ Although LNCs are generally biocompatible, long-term toxicity information is scarce, and the risk of immunogenicity or off-target effects cannot be excluded, particularly with functionalized or cationic formulations. Thus, it is essential to carefully optimize surface chemistry, dosage regimens, and in vivo safety profiling to advance LNC-based therapies.^141^ The cytotoxicity of LNCs occurs in a dose-dependent manner, especially when incorporated with cationic lipids or surfactants like DOTAP or high levels of polysorbates, which have the potential to destabilize cell membranes and cause unwanted toxicity towards non-cancerous cells. Oxidative stress and mitochondrial damage have also been described following extended exposure to some lipid formulations, which may trigger apoptosis in off-target tissue. Immune-mediated toxicities like complement activation, cytokine release, and hypersensitivity reactions have been reported to occur, notably with PEGylated or surface-functionalized LNCs. Inflammation can also be induced upon recognition by pattern recognition receptors, resulting in acute or chronic toxicity. Toxicity can systemically occur upon accumulation in organs such as the liver and spleen through the RES, especially for big or surface-charged particles.^142,143^

## Conclusion and Future Perspectives

 LNCs offer a novel and promising delivery system for therapeutic medicines in cancer treatment. These nanocarriers provide a special combination of characteristics that increase their effectiveness and adaptability in oncological applications by combining the benefits of polymeric nanoparticles and liposomes. LNCs present certain advantages over the rest of LNPs due to their inherent structural and functional features. Compared to conventional LNPs or liposomes, LNCs are constructed with a core-shell architecture, where hydrophobic drugs are encapsulated in the lipid core, and hydrophilic drugs are transported in the hydrophilic shell. This biform functionality gives them additional flexibility in drug delivery. The phase inversion process to prepare LNCs guarantees higher stability, allowing efficient drug protection and targeted delivery. LNCs also offer enhanced bioavailability and pharmacokinetics, resolving some of the significant issues of conventional chemotherapeutics, including poor solubility, quick systemic clearance, and non-specific distribution. Through improved drug solubility, extended circulation time, and controlled release, LNCs enhance drug concentration in tumor sites and reduce systemic toxicity. These distinct features render LNCs a more flexible and efficient option in cancer treatment than other LNPs. By making hydrophobic drugs more soluble, extending their circulation time, and enabling controlled release, LNCs solve these problems and increase the concentration of drugs at the tumor site while lowering systemic toxicity.

 In the future, improvements in genomics and molecular biology will further develop the potential of LNCs for personalized medicine to allow more targeted and individualized therapeutic approaches for cancer therapy. Individual genetic profiles will be catered for in therapies, and leukemia cells will be made to carry customized drug combinations that target specific disease pathways. Since LNCs have an enhanced ability to deliver combinational drugs, this strategy is very effective in customized drug combinations for personalized medicine. A practical approach to cancer treatment is provided by creating multifunctional LNCs that simultaneously deliver many therapeutic agents, such as immunomodulators, chemotherapeutics, and gene therapies. Theragnostic, which combines therapeutic and diagnostic capabilities in LNCs, will optimize treatment regimens by enabling real-time drug distribution and therapeutic response monitoring. LNCs can transform cancer treatment by utilizing technology breakthroughs and tackling present issues, ultimately improving patient outcomes and progressing the oncology sector.

## Competing Interests

 The authors declare that they have no known competing financial interests or personal relationships that could have appeared to influence the work reported in this paper.

## Ethical Approval

 This study did not involve human participants or animals.
